# Intra-arterial PSMA injection using hepatic arterial infusion pump in intrahepatic cholangiocarcinoma: a proof-of-concept study

**DOI:** 10.1186/s41747-024-00496-4

**Published:** 2024-08-01

**Authors:** Mara Marieke Katrien Veenstra, Erik Vegt, Marcel Segbers, Stijn Franssen, Bas Groot Koerkamp, Frederik Anton Verburg, Maarten Guillaume Josephus Thomeer

**Affiliations:** 1 Department of Radiology & Nuclear Medicine, Erasmus MC University Medical Centre Rotterdam, Rotterdam, The Netherlands; 2 Department of Surgery, Erasmus MC University Medical Centre Rotterdam, Rotterdam, The Netherlands

**Keywords:** Cholangiocarcinoma, Gallium 68 PSMA-11, Injections (intra-arterial), Positron emission tomography-computed tomography, Precision medicine

## Abstract

**Abstract:**

Prostate-specific membrane antigen (PSMA) targeted tracers show increased uptake in several malignancies, indicating a potential for peptide radioligand therapy. Intra-arterial injection of radiotracers can increase the therapeutic window. This study aimed to evaluate the feasibility of intra-arterial injection of [^68^Ga]Ga-PSMA-11 for intrahepatic cholangiocarcinoma and compare tracer uptake after intrahepatic arterial injection and intravenous injection. Three patients with intrahepatic cholangiocarcinoma received [^68^Ga]Ga-PSMA-11 through a hepatic arterial infusion pump, followed by positron emission tomography/computed tomography (PET/CT). Two-three days later, patients underwent PET/CT after intravenous [^68^Ga]Ga-PSMA-11 injection. All tumours showed higher uptake on the intra-arterial scan compared with the intravenous scan: the intra-arterial / intravenous standardised uptake value normalised by lean body mass ratios were 1.40, 1.46, and 1.54. Local intra-arterial PSMA injection is possible in patients with intrahepatic cholangiocarcinoma. Local injection increases tumour-to-normal tissue ratios, increasing the therapeutic window for theranostic applications.

**Relevance statement:**

Intra-arterial Prostate specific membrane antigen (PSMA) injection increases the therapeutic window for potential theranostic application in intrahepatic cholangiocarcinoma.

**Key Points:**

- Three patients with intrahepatic cholangiocarcinoma underwent PET/CT after intra-arterial and intravenous injection of [^68^Ga]Ga-PSMA-11.
- Intra-arterial injection showed higher uptake than intravenous injection.
- PSMA-targeted imaging could be valuable for a subset of intrahepatic cholangiocarcinoma patients.

**Graphical Abstract:**

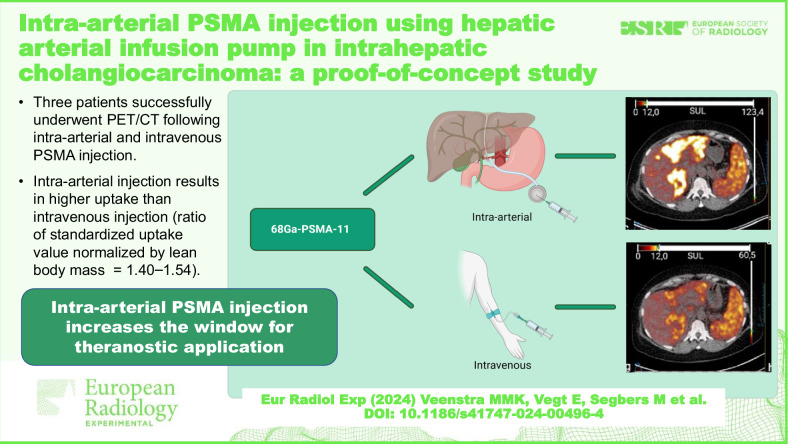

## Background

Prostate-specific membrane antigen (PSMA) is a transmembrane protein that is overexpressed in prostate carcinoma, but also in several other tumours, among which intrahepatic cholangiocarcinoma (ICC) [1, 2]. ICC is the second most common type of hepatobiliary cancer and has a poor prognosis, with 5-year survival rates of less than 10% [3, 4]. Most patients present with advanced disease without possibility of resection, stressing the clinical need for new therapeutic options.

Studies have shown PSMA-expression in 87% (13/15) [5] and 43% (48/111) [6] of ICCs during histological evaluation. Literature regarding PSMA uptake in ICC on positron emission tomography (PET) is limited to case reports with incidental findings where PSMA was used for prostate cancer [7, 8]. Studies primarily assessing PSMA PET in ICC are lacking. An important reason to pursue PSMA-targeted imaging of ICC is the prospect of PSMA-targeted peptide radioligand therapy (*e.g*., ^177^Lu-PSMA-617) for patients with ICC with high PSMA expression.

Intra-arterial tracer administration near a tumour may provide a higher tumour uptake than intravenous injection, with similar or lower normal tissue uptake due to the first-pass effect, increasing therapeutic width, and is increasingly being investigated [9]. Whether intra-arterial tracer injection could be of significance for ICC remains unknown.

This study aimed to evaluate intra-arterial injection feasibility and standardised uptake values of ICC with [^68^Ga]Ga-PSMA-11 PET/CT (computed tomography) and to compare these values between local intra-arterial injection through a hepatic arterial infusion pump and intravenous injection.

## Methods

**Fig. 1:**
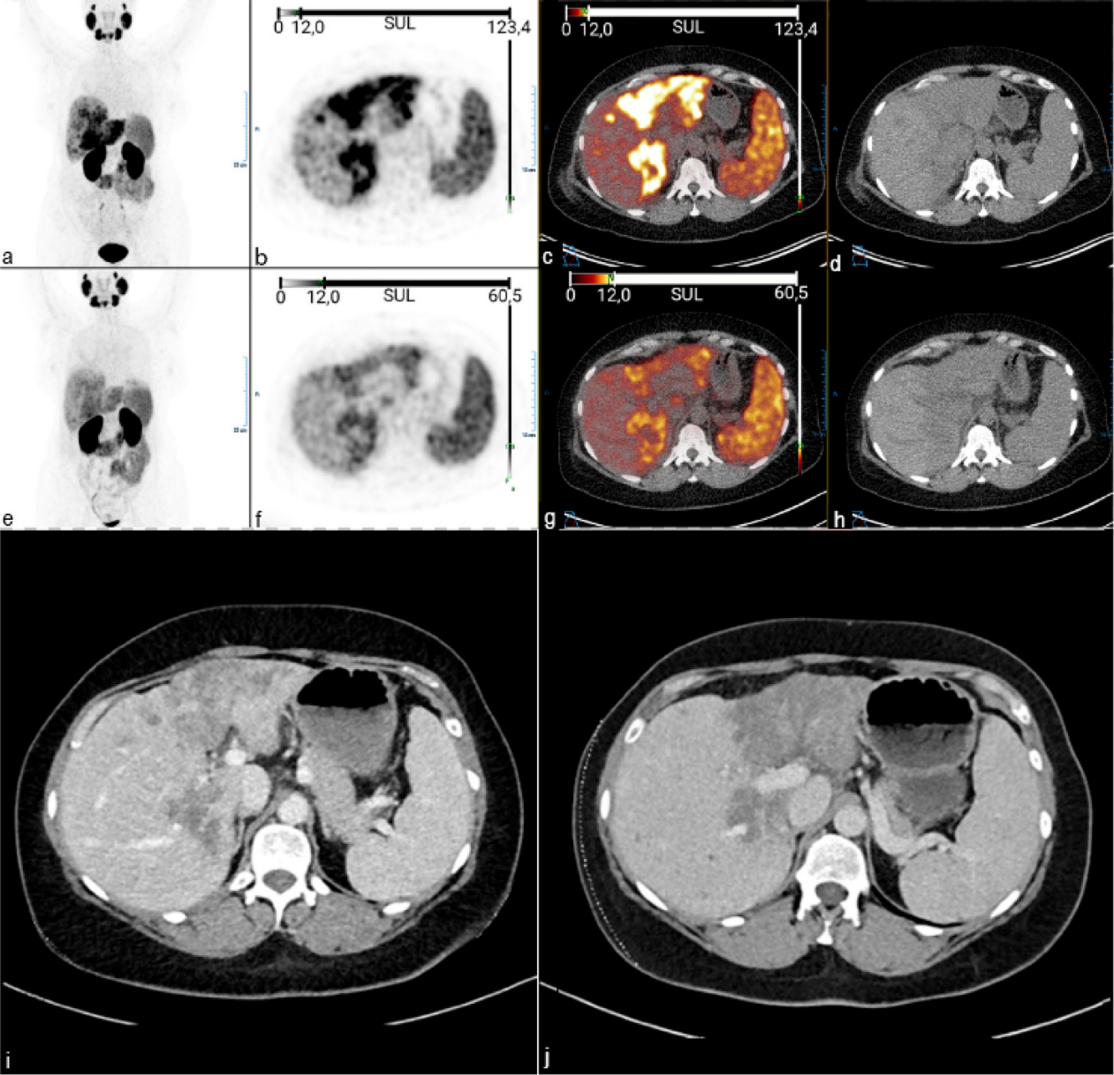
Patient 2, a 38-year-old female with inoperable intrahepatic cholangiocarcinoma in segments II, III, IV, V and VI. Intra-arterial (**a**–**d**) and intravenous (**e**–**h**) [^68^Ga]Ga-PSMA-11 PET/CT images are shown. Intra-arterial infusion yielded higher uptake than intravenous injection. Coronal maximum intensity projection (**a,** **e**), axial PET (**b,** **f**), fused PET/CT (**c,** **g**), CT (**d,** **h**), CT six weeks prior to PET/CT (**i**) and three months after PET/CT (**j**). CT, Computed tomography; PET, Positron emission tomography; PSMA, Prostate specific membrane antigen

This study is a side study of the PUMP II trial (EudraCT number 2018-004013-41) [10]. This trial included patients with pathologically proven inoperable ICC who were scheduled for placement of a hepatic arterial infusion pump for local intrahepatic chemotherapy. The study was performed in line with the principles of the Declaration of Helsinki. Approval was granted by the Ethics Committee of the Erasmus MC University Medical Centre, Rotterdam (2019-11-27/NL70452.078.19), and all subjects signed an informed consent form. This proof-of-concept study included patients participating in the PUMP II trial. The authors affirm that the participants provided informed consent for the publication of their data and the images in Fig. 1.

The hepatic arterial infusion pump was placed in the gastroduodenal artery according to the PUMP study protocol [11]. Patients routinely underwent CT-abdomen and ^99m^Tc-macro aggregated albumin scintigraphy to confirm the correct placement of the pump, correct arterial perfusion and the absence of extrahepatic perfusion. After this, participants of this study received [^68^Ga]Ga-PSMA-11 (1.5 MBq/kg body weight) via the pump, followed by flushing with 10 mL of saline. A heparin lock (10 mL) was administered to the pump to prevent intra-pump coagulation. PET/CT was acquired 60 min after injection, starting with a low-dose CT. PET/CT was performed from the skull to the thighs (3 min per bed position, slice thickness 3 mm) on a Biograph mCT 128 (Siemens Healthineers, Erlangen, Germany). PET images were reconstructed in compliance with EANM Research Ltd. (EARL)-2 standards [12]. After the tracer had fully decayed, patients would undergo PET/CT with intravenous [^68^Ga]Ga-PSMA-11 (1.5 MBq/kg body weight) using the same protocols.

All intra-arterial and intravenous PET/CT images were reviewed by the same nuclear medicine physician (E.V.). Outcomes were expressed using standardised uptake values normalised by lean body mass (SUL). SUL was calculated using the James equation [13]. Regions of interest were drawn using the Vue Picture Archiving and Communication System software (Philips, Amsterdam, the Netherlands). Regions around the tumour and the left parotid gland were drawn with an isocontour of 40% of SUL_max_. An area of normal looking liver tissue, approximately 2.8 cm in diameter, was selected to measure SUL in normal liver tissue. SUL_peak_ was measured in tumour tissue. In normal liver tissue and the left parotid gland, SUL_mean_ was also measured.

The primary outcome was the ratio of tumour SUL after local intra-arterial *versus* intravenous injection. Secondary outcomes were the feasibility of local intra-arterial [^68^Ga]Ga-PSMA-11 injection, [^68^Ga]Ga-PSMA-11 biodistribution, and salivary gland SUL upon local intra-arterial *versus* intravenous injection. Outcomes are shown using ratios comparing intra-arterial and intravenous uptake values. Due to the small number of patients, no statistical analyses were performed.

## Results

Three patients with a hepatic arterial infusion pump underwent PET/CT after intra-arterial and intravenous [^68^Ga]Ga-PSMA-11 injection (Table 1). For all patients, ^99m^Tc-macro aggregated albumin scintigraphy showed lung shunt fractions smaller than 1.5%. All [^68^Ga]Ga-PSMA-11 injections were performed without complications.

**Table 1:** Patient characteristics

Patient number	Age (years)	Sex	Tumour characteristics^*^	Previous treatment
1	76	Male	Hypovascular, 7 cm, partially neuroendocrine differentiation	Bile duct stenting
2	38	Female	Hypovascular, 8.5 cm with multiple very small (< 5 mm) peritoneal and lung metastases, mild differentiation	Gemcitabine and cisplatin
3	29	Female	Hypovascular, 5.2 cm	Bile duct stenting

* Appearance on computed tomography, diameter and pathology (if available)

ICCs showed [^68^Ga]Ga-PSMA-11 uptake for both injection methods (Fig. 1). All tumours showed higher SULs on the intra-arterial scan compared with the intravenous scan (intra-arterial SUL_peak_ / intravenous SUL_peak_ ratios 1.40–1.54, Table 2). Very small metastases (< 5 mm in diameter) did not show uptake. Uptake in normal liver tissues was similar between intra-arterial and intravenous scans (intra-arterial SUL_peak_ / intravenous SUL_peak_ ratios 0.86–1.3). Uptake in the salivary glands was similar to slightly lower after intra-arterial injection (Table 2).

**Table 2:** PET/CT outcomes

	Patient 1			Patient 2			Patient 3		
	IA	IV	Ratio*	IA	IV	Ratio*	IA	IV	Ratio*
Tumour									
SUL_peak_	11.6	8.3	1.40	10.2	7.0	1.46	9.4	6.1	1.54
Metastases	NA	NA	NA	Negative	Negative	NA	NA	NA	NA
Liver									
SUL_peak_	3.7	4.3	0.86	3.0	2.9	1.03	2.7	2.4	1.13
SUL_mean_	3.5	3.6	0.97	2.5	2.6	0.96	2.2	2.1	1.05
Left parotid									
SUL_peak_	12.9	13.0	0.99	11.0	12.2	0.90	9.2	10.3	0.89
SUL_mean_	9.5	9.8	0.97	8.0	8.7	0.92	6.8	7.6	0.89

* Ratio: IA/IV. *IA* Intra-arterial injection, *IV* Intravenous injection, *SUL* Standardised uptake value normalised by lean body mass, *NA* Not applicable

Patient 1 had areas that showed increased [^68^Ga]Ga-PSMA-11 uptake around dilated bile ducts in normal liver tissue. SUL_peak_ was 20.6 on the intra-arterial PET and 10.9 on the intravenous PET (ratio 1.9). Both intra-arterial and intravenous PET/CT in patient 2 showed diffusely increased [^68^Ga]Ga-PSMA-11 uptake in large parts of the liver, which seemed more extensive than that on the recent contrast enhanced CT-scan from six weeks prior. Interestingly, CT three months post-PET/CT showed tumour progression within these segments (Fig. 1). Patient 3 had diffusely increased tracer uptake in tumour tissue.

## Discussion

This proof-of-concept study showed that it was feasible to perform injection of [^68^Ga]Ga-PSMA-11 in the hepatic artery, and that tracer uptake was higher after intra-arterial injection than after intravenous injection.

PSMA uptake on PET in cholangiocarcinoma has been reported in a few case-reports [7, 8]. This study showed PSMA uptake in all patients, supporting these findings. In addition to diagnostic use, PSMA-targeted imaging of ICC may have theranostic potential in combination with PSMA-targeted peptide radioligand therapy (*e.g*., ^177^Lu-PSMA-617) in patients with high tumour PSMA expression.

This study showed that it is possible to administer [^68^Ga]Ga-PSMA-11 locally through intra-arterial injection using a hepatic arterial infusion pump. Potential advantages of a more local tracer administration are higher uptake in areas of interest and lower uptake in other organs due to the first-pass effect, potentially increasing the therapeutic window. Due to the physiological uptake of PSMA in tissues such as salivary glands and bone marrow, dry mouth and haematological toxicity are common side effects of PSMA-targeted therapy [14]. Increasing the therapeutic window could improve tumour control and decrease side effects, improving patient health-related quality of life. This study showed that intra-arterial PSMA injection did not cause higher tracer uptake in other organs when compared with intravenous injection. PSMA uptake in normal liver tissue also did not increase, likely explained by the liver’s predominantly portal venous blood supply.

One of our patients showed high [^68^Ga]Ga-PSMA-11 uptake around areas with dilated bile ducts, possibly caused by inflammation of these bile ducts. This could be a disadvantage for the use of PSMA-targeted peptide radioligand therapy in ICC as dilated bile ducts and/or cholangitis are common in this disease. High tracer uptake could possibly result in bile duct damage and bile leaks, which could cause inflammation and infection. The possibility of PSMA-targeted peptide ligand therapy might, therefore, be limited to patients with ICC that show no signs of bile duct dilatation. More research is needed to assess the full effect of PSMA on dilated bile ducts. It would be interesting to see if other tracers, i.e. targeting integrin αvβ6 that shows potential for its use in cholangiocarcinoma [15], yield better results.

In patient 2, both intra-arterial and intravenous [^68^Ga]Ga-PSMA-11 PET/CT showed tumour extension that could not yet be seen using CT. This implies that PSMA expression might be an earlier sign of neoplastic neovascularisation than contrast enhancement. If [^68^Ga]Ga-PSMA-11 PET/CT has the potential to detect progressive disease earlier than the imaging techniques currently in use, [^68^Ga]Ga-PSMA-11 PET/CT could be an important opportunity to optimise treatment for patients with ICC and warrants further research.

As a proof-of-concept study, the number of patients included is small. This cohort size could limit the reliability of extrapolation of our results. Though differences in SUL were found between intra-arterial and intravenous injection, it is unclear whether these differences are substantial enough to be clinically relevant. Patients had differences in tumour size, differentiation and the extent of disease, which might have influenced results. Two patients were relatively young, potentially resulting in disease mechanisms (*i.e*., congenital diseases) that differ from the general ICC patient population. All tumours in this cohort were hypovascular, whilst PSMA uptake has been associated with neo-angiogenesis [16, 17]. Therefore, tracer uptake might be higher in hypervascular ICC.

Recent studies have shown the possibility to use PSMA in cancers other than prostate cancer [1, 2]. In a disease such as cholangiocarcinoma it remains important to highlight new areas for imaging and treatment. Local intra-arterial PSMA injection is feasible in patients with ICC. The increased therapeutic window of intra-arterial injection compared with intravenous injection could be an interesting incentive to explore the possibility of PSMA-targeted peptide radioligand therapy for a subset of ICC patients.

## Data Availability

The datasets generated during and/or analysed during the current study are available from the corresponding author on reasonable request.

## Abbreviations

CT: Computed tomography
ICC: Intrahepatic cholangiocarcinoma
PET: Positron emission tomography
PSMA: Prostate specific membrane antigen
SUL: Standardised uptake values normalised by lean body mass
